# Reciprocal Relationships Between Parental Involvement and Academic Performance in Early Adolescence: A Two-Year Longitudinal Study in China

**DOI:** 10.1007/s10964-024-02102-7

**Published:** 2024-10-26

**Authors:** Yi Yang

**Affiliations:** Academy of Wellness and Human Development, Faculty of Arts and Social Sciences, Hong Kong Baptist University, Kowloon, Hong Kong

**Keywords:** Gender, Parental involvement, Academic performance, Early adolescence, China, Reciprocal relationship

## Abstract

Previous literature has focused on the overall influence of parental involvement on adolescents’ academic performance, while less attention has been accorded to the reciprocal relationships between different forms of parental involvement and academic performance across gender. The present study examined the reciprocal associations between different forms of parental involvement and adolescents’ academic performance using nationally representative data. A total of 9449 Chinese adolescents (47.82% girls, *M*_age_ = 13.21, *SD* = 0.65, 91.20% Han Ethnicity) have participated in the two-year and two-wave longitudinal study. Cross-lagged model results indicated reciprocal associations between parent-reported involvement and academic performance. Autonomy-supportive parental involvement in Grade 7 was positively associated with better academic performance in Grade 8, while behavioral control was negatively associated with later academic performance. Parental socialization practices vary by adolescent’s gender. Compared with girls, boys experienced increased parental behavioral control in response to better academic performance. The findings reflect the persistence of stereotypical gender expectations and gender socialization in contemporary China.

## Introduction

Early adolescence, roughly from the age of 12–16, is an accelerating development period beginning with the onset of puberty. The spurt of bioecological and psychological development leads to dramatic changes in adolescents’ behaviors and relationships with significant others. Young adolescents begin to strive for autonomy and resist parental authority as neural changes make adolescents seek greater independence and status (Dahl et al., 2018). This change may evoke more conflicts between parents and young adolescents and make parental involvement more challenging. Although cultural tradition believes the influence of parents is declining and peer influence is starting to manifest, recent empirical research has provided compelling evidence that parenting processes still weigh more for adolescents’ development and well-being than peer processes (Dahl et al., 2018). Parental involvement plays a paramount role in young adolescents’ academic performance, socio-emotional development, and well-being (Barger et al., 2019). Compared with children in late adolescence, children in early adolescence whose academic performance tended to be influenced more by negative parental involvement, e.g., high parental pressure and control (Starr & Riemann, 2022). This indicates that the degree to which parental involvement makes a difference is influenced by the characteristics of children, e.g., children’s developmental phase. Yet, most extant studies focus on how parental involvement influences children while neglecting the reciprocal relationships between parental involvement and children’s academic performance (Hong et al., 2010). The influence of different forms of parental involvement on children’s academic performance may also vary by culture. In addition, meta-analysis studies found mixed findings on how children’s characteristics, such as gender, influenced children’s experience of parental involvement and its influence on academic performance. The current study examines the extent to which different forms of parental involvement are reciprocally associated with young adolescents’ academic performance and whether such associations vary by gender in contemporary China.

### Parental Involvement as a Multifaceted Construct Predicting Academic Performance

Parental involvement is generally defined as parents’ or caregivers’ investment in their children’s education and learning or “parental participation in the educational processes and experiences of their children” (Jeynes, 2010, p. 62). Parental involvement is a multifaceted, not unidimensional construct, representing a wide range of parenting practices, including parents’ educational expectations, communication with children about school-related matters, parental supervision, and school contact and participation (Fan & Chen, 2001). A recent review further identified that parents’ value of education and educational trips to the library or museum are essential forms of parental involvement (Boonk et al., 2018). Yet, there is also dissensus whether parents’ cognitions (e.g., educational expectation and value of education) should be considered as parental involvement as they do not represent parents’ actual behaviors and investment (Barger et al., 2019).

Although extant studies generally reported a significant relationship between parental involvement and academic performance with a small to medium effect size, there are inconsistent findings on which forms of parental involvement are more predictive of better academic performance (Barger et al., 2019). The magnitude of association varies when parental involvement is measured globally or examined by specific aspects of parental involvement (Boonk et al., 2018). As mentioned, when parental involvement is globally measured, it seems closely related to students’ learning performance. The association appears insignificant or even negative when parental involvement is narrowly defined as academic control and homework supervision (Strayhorn, 2010). The yet conclusive findings reflect how multifaceted and complex the parental involvement construct is and the deficiency of comprehensive measures. Although the multidimensionality of parental involvement has been widely recognized, many extant studies treat it as a broad construct when examining its association with adolescents’ academic performance (Boonk et al., 2018). Either used a general composite to measure parental involvement (Koepp et al., 2022; Xiong et al., 2021) or did not examine the influence of specific forms of parental involvement (Cheung & Pomerantz, 2012; Hsu et al., 2011). Thus, the extent to which different forms of parental involvement can uniquely contribute to adolescents’ academic performance, or which does not matter as long as parents get involved, remains yet comprehensive across socio-cultural contexts.

### From a Transactional Perspective to Understand the Relationship between Parental Involvement and Academic Performance

Most studies generally have assumed that parental involvement is predictive of academic performance. Yet, the direction of the relationship remains unclear because most studies are cross-sectional in nature, which cannot build up temporal order and determine whether parental involvement precedes or responds to students’ academic performance (Hong et al., 2010). There seems to be scant attention on the reversed association: how children influence parents and how parents interact with them. According to the most recent comprehensive review, among all the 31 studies that examined the association between parental involvement and adolescents’ academic performance, only seven were longitudinal-designed, and two examined the bidirectionality (Boonk et al., 2018).

The transactional theory model considers that “development of any process in the individual is influenced by interplay with processes in the individual’s context over time” (Sameroff, 2009, p. 6). It emphasizes the bidirectional and interdependent relationship between the individual and the environment. Individual actively constructs and organizes his/her growth where the environment is plastic to the individual’s reaction. For example, children with more problematic behaviors may solicit higher levels of parental involvement with more restrictions (Wang et al., 2011). Similarly, parents may react differently based on their children’s academic performance. Thus, it is imprudent to assume a parent-driven and unidimensional relationship between parental involvement and adolescents’ academic performance with cross-sectional data. Instead, given the nature of the transactional relationship between individual and experience, using longitudinal datasets can address the bidirectional relationship between parental involvement and students’ academic performance. Furthermore, using nationally representative data with abundant demographic information can account for the influence of broader socioeconomic conditions at the community- and family level on adolescents’ academic performance (Ruiz et al., 2018) and their parent’s involvement (Bhargava & Witherspoon, 2015).

### Parental Involvement and Academic Performance across Contexts

When talking about the role of culture in human development, there are some fundamental similarities in human development processes regardless of socio-cultural context. As mentioned, studies across countries generally demonstrated that parental involvement is closely related to adolescents’ academic performance (Boonk et al., 2018). Taking parents’ behavioral control as an example, cross-cultural comparison indicated that parents’ behavioral control, including restricting children’s behaviors and soliciting their whereabouts, benefited 7^th^ graders’ examine performance both in the United States and China (Wang et al., 2007).

Meanwhile, cultural dissimilarities are also observed given the unique cultural heritage and socialization across socio-cultural contexts. For example, some studies in Asian contexts found that parents’ monitoring of homework supervision was positively associated with young adolescents’ academic performance in contrast to the findings in Western contexts (Park et al., 2011). It was found that despite behavioral control benefiting both children from European and Asian backgrounds, European children benefited more from their parents’ autonomy-supportive behaviors, e.g., opinion exchange and school-related discussion and encouragement (Wang & Sheikh‐Khalil, 2014). Similarly, one cross-culture study found that granting children decision-making choice was associated with better school engagement in the United States but not in the collectivist-oriented Ghana (Marbell‐Pierre et al., 2019). These cultural dissimilarities may lie in the differences in the ways that parents are involved in their children’s lives and how children perceive such involvement. In Confucian culture, parents represent the family authority, for which there is always a distance from their children even though parents sacrifice themselves for their children’s future development. Thus, traditional Chinese parenting is characterized by controlling and having less room for equal discussion. In return, children should show their filial obedience without any doubt. Under such cultural norms, children may anticipate parents’ high levels of control and low levels of autonomy and respond better (Cheung & Pomerantz, 2012). Yet, such a static portrayal of Chinese parenting seems cliché and questioned. With globalization and rapid societal changes, many Chinese parents have integrated autonomy-supportive approaches while retaining certain levels of psychological and behavioral control. If adolescents perform well in school, Chinese parents will grant them greater autonomy (Wang et al., 2012).

When it comes to academic performance, Chinese parents put great emphasis on their children’s academic excellence and believe in the importance of rearing. In the traditional Chinese culture, children’s performance reflects on and achieves for the whole family. Children’s academic performance is viewed as a collective family responsibility rather than individual effort. Chinese parents’ feeling of fulfillment and worth is greatly contingent on their children’s performance at school (Ng et al., 2014). Therefore, Chinese parents are motivated to take a very proactive role in children’s academic learning, regardless of occupation or educational level. They tend to be actively involved in children’s learning at home by helping and supervising homework and monitoring activities that may interfere with their academic performance (Wang et al., 2007). In contrast, Chinese parents were less likely to initiate education-relevant discussions with their children. According to the PISA survey, only 40% of surveyed Chinese parents talked about school life with their children, while the average for OECD countries was 79% (Borgonovi & Montt, 2012). In addition, Chinese parents do not typically seek frequent personal connections with teachers or regularly engage in school activities, e.g., contact teachers and attend parent-teacher meetings (Huntsinger & Jose, 2009). The close supervision and the clear line between home and school reflect traditional Chinese-featured parental involvement: a defined separation between teachers and parents.

Prior studies extensively discussed the control and supervision aspects of Chinese parental involvement. In contrast, school-related parental involvement was less investigated and was found to have less impact on children’s academic performance as Chinese parents were generally less involved in schools (Lau et al., 2011). With the extensive use of instant message tools in recent years, school contact has become more frequent. Thus, Chinese parents are more involved in school activities than before (Gong et al., 2021). However, it is still unclear whether the changing landscape in parental involvement will influence children’s academic performance differently.

### Moderating Role of Gender

As indicated above, parental involvement in children’s learning is a cultural practice. It is also a practice that reflects gender socialization. Parents were involved in their children’s learning regardless of the gender of their children, while significant variations were observed in the forms of parental involvement for their daughters and sons (Borgonovi & Montt, 2012). Extant studies indicated that parents were generally more likely to discuss school life with girls than boys (Silinskas & Kikas, 2019). For boys, parents tended to be involved through frequent communications with schools and behavioral control, e.g., homework checking and limit setting (Borgonovi & Montt, 2012). Such differences may reveal that parental involvement is a reciprocal process. Parents determine and adjust their parenting practices based on their children’s gender. It is suggested that teenage boys generally exhibit less persistence and poorer self-regulation than girls, compelling their parents to be more controlling and communicate with teachers more frequently to monitor their school performance (Silinskas & Kikas, 2019).

On the one hand, from a parent-driven perspective, more parental involvement in girls’ learning through discussion with school life may represent greater autonomy support parents grant to girls. On the other hand, it could be the representation of traditional gender socialization that girls are more dependent than boys. The higher level of behavioral control and frequent communication with schools may also relate to parents’ higher educational and career expectations for sons than daughters. In traditional Chinese culture, old parents need to rely on their sons, so they restrict autonomy to sons for better academic and career futures (Ng et al., 2014).

In addition to gender variations in the forms of parental involvement, parental involvement has exhibited both similar and varied influences on boys and girls during adolescence. When examining parental involvement as a general construct, it is found that boys and girls both benefited from parental involvement in their learning with boys slightly benefiting more from parental involvement (Jeynes, 2010). When it comes to the specific forms of parental involvement, extant studies show inconsistencies in the findings. One study conducted in Estonia found that boys who perceived higher levels of parental control in their math homework were negatively influenced, which was not the case for girls (Silinskas & Kikas, 2019). On the contrary, one study with Latino families indicated that harsh and average levels of control benefited Latino boys’ academic performance but it worked contrariwise for girls (Camacho-Thompson et al., 2019). The mixed findings suggest that the specific associations between different forms of parental involvement and academic performance across gender may be sensitive to the cultural context.

## The Current Study

**Fig. 1:**
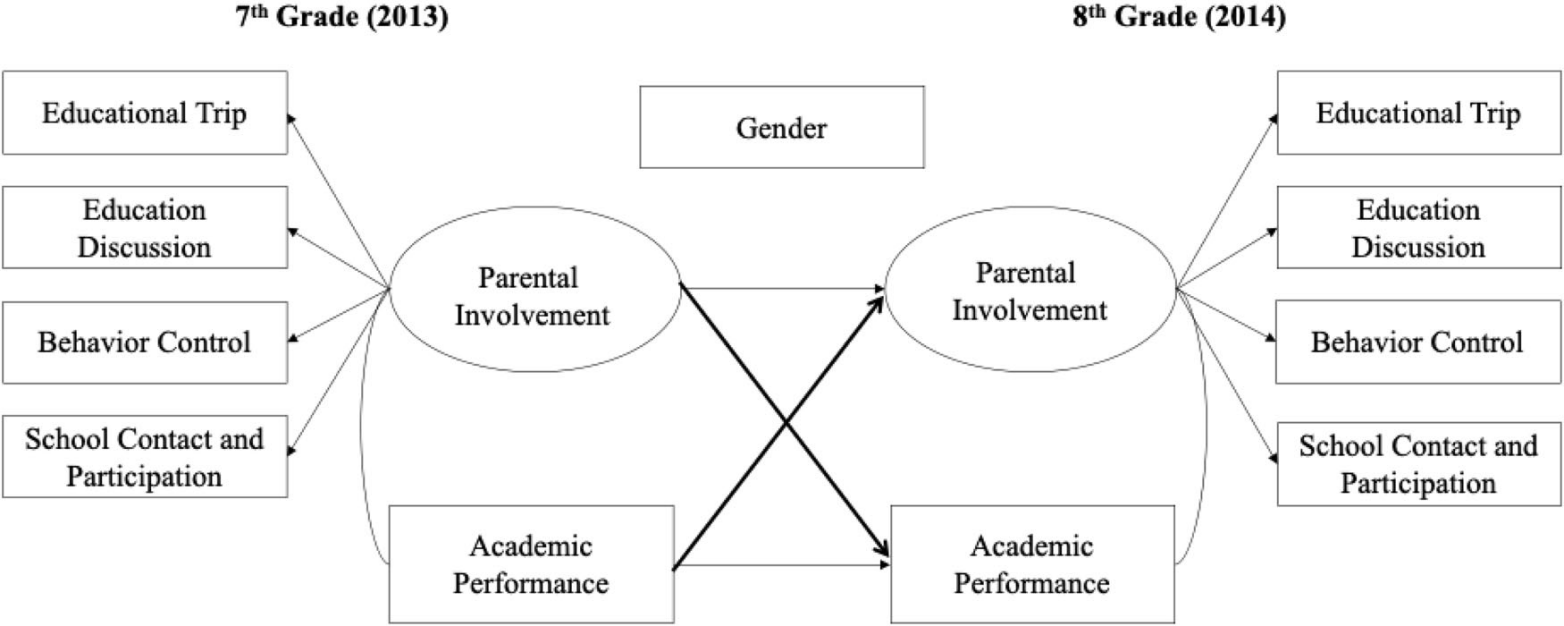
Theoretical Model of Parental Involvement and Academic Performance. *Note*. Bolded cross-lagged paths correspond to the hypothesis

The role of parental involvement in adolescents’ academic performance has been widely recognized, while less attention has been accorded to the role of adolescents’ gender and their academic performance in soliciting different forms of parental involvement. The present study addresses the research gap in the literature by examining the two research objectives: (1) the extent to which different forms of parental involvement are reciprocally associated with Chinese adolescents’ academic performance and (2) whether gender moderates the associations. Given the transactional nature of individual development, it was hypothesized that different forms of parental involvement in 7th grade would influence young adolescents’ academic performance in 8th grade differently, and adolescents’ prior academic performance would influence parents’ strategies of parental involvement. As previous studies have yielded mixed findings of gender difference across contexts, the current study has no specific hypothesis on the direction and effect sizes of the variations but hypothesizes that specific associations may differ by gender. The conceptual framework of the study is illustrated in Fig. 1.

## Method

### Data and Participants

This study utilizes data from the China Education Panel Survey (CEPS). The CEPS is a large-scale, longitudinal, nationally representative annual survey conducted to understand how family, school, and community factors have influenced student learning outcomes since 2013. The baseline survey was conducted from 2013 to 2014, with one follow-up from 2014 to 2015. CEPS employed a multistage probability proportionate to size sampling approach to ensure population-representativeness. The sampling was conducted in three stages: (i) randomly selecting 28 county-level administrative units (county, districts, city); (ii) sampling four lower secondary schools in each selected county-level administrative unit; (iii) sampling two 7th grade and two 9th grade classes in each selected lower secondary school; (iv) recruiting all students and their parents in the selected classes. At each wave, questionnaires were directly distributed to students, parents, school principals, and local community officers. Information on students’ midterm standardized test scores was obtained from school records. In addition to the mid-term test grades provided by schools, students also took up a standardized cognitive ability test designed by the National Survey Research Center (NSRC) at Renmin University of China. A sample of 10,279 7th-grade and 9208 9th-grade students from 438 classes of 112 were surveyed in the baseline survey. The follow-up survey in 2014–2015 tracked 9449 8th graders (7th graders in the baseline survey) with a 91.9% successful follow-up rate. The attrition was mainly due to the school transfer and dropout. The final sample included 9449 students who participated in baseline and follow-up surveys, among whom 52.2% were boys. Ethics approval was obtained from Renmin University of China, and consent was obtained from all participants before the data collection.

### Measures

#### Parental involvement

Parent-reported parenting practices were used to describe parental involvement. To have a holistic depiction of parental involvement, this study intends to capture a wide range of parental involvement activities. Although some studies consider educational expectation as one form of parental involvement, this study excluded educational expectation since it does not denote actual practice and investment (Barger et al., 2019). Based on the extant reviews (Barger et al., 2019; Boonk et al., 2018; Fan & Chen, 2001), four forms of parental involvement were selected: Educational Trips (2 items), Education Discussion (4 items), Behavioral Control (6 items), and School Contact and Participation (5 items). Educational Trips concerns family visits to the library and museum or other cultural and learning-related participation, for example, watching sports or music shows. Parents responded to the items on a six-point Likert Scale (1 = never, 2 = once a year, 3 = once every half year, 4 = once a month, 5 = once a week, 6 = more than once a week). Education Discussion refers to the parent-child discussion about school life, such as school activities and interpersonal relationships, on a three-point scale (1 = never, 2 = sometimes, 3 = often). Behavioral Control measures the extent to which parents restrict children’s behaviors, for example, TV rules, homework supervision, and other limit-setting on a three-point scale (1 = I don’t care, 2 = I care but not strict, 3 = I’m very strict). School Contact and Participation indicated the frequency and content focus of teacher-parent communication initiated by the parents on a four-point scale (1 = never, 2 = once, 3 = two to four times, 4 = five times and above). A higher score indicates a higher level of parental involvement. The Cronbach’s Alpha coefficients were 0.79 and 0.81, respectively, for the 17 items of parental involvement at two waves of data collection, suggesting good reliability.

#### Adolescent’s academic performance

Adolescents’ midterm grades were obtained directly from school records. The sum of standardized Chinese, English, and Mathematics test scores across each wave was used to indicate adolescents’ overall academic performance. These scores ranged from 55.49 to 257, with higher scores denoting better academic performance. Standardized Cognitive Ability Test (CAT) scores were also calculated for the sensitivity analysis, given their national comparability compared to midterm grades. The CAT, comprising 20 items designed for 7th-grade students and 35 items for 8th-grade students, assesses three key learning domains: language, geometry and numeracy, and logical inference. The test demonstrated adequate internal consistency, with a Cronbach’s alpha of 0.69. To compute students’ cognitive abilities, the raw CAT scores were analyzed using the three-parameter Logistic (3PL) Item Response Theory model, which rules out the possibility of guessing and thereby provides a more accurate reflection of students’ cognitive abilities. The correlation between students’ midterm standard test scores and cognitive ability tests is 0.95, indicating a good concurrent validity.

#### Demographic Covariates

Participants were asked to report their individual-, family-, school-, and community-level demographic information.

##### Student characteristics

Students’ gender, ethnicity, and whether the only-child family was dummy coded, for example, 1 = girl, 0 = boy.

##### Family characteristics

***Living Status:*** 1 = at least one of the parents does not live with the child, 0 = children live with parents.

***Family SES:*** The information on family SES was collected through students’ self-reported questionnaire at Time 1, which was composed of four variables: maternal education, paternal education, and parents’ occupation. Parents’ education was coded into nine categories from 1 = no formal education to 9 = postgraduate and above. Parents’ occupations were also coded into nine categories from 1 = unemployed or laid-off workers to 9 = government officials, staff of public institutions, and civil servants. The average standardized scores of four variables were used as the index of family SES.

##### School characteristics

***School Type:*** Reported by school leaders. 1 = public school, 0 = private school.

***School Ranking:*** School leaders report the school quality ranking in local districts. 1 = the lowest to the middle tier, 2 = the upper middle tier, 3 = the highest tier.

##### County characteristics

***Place of residence:*** 1 = urban residence, 0 = rural residence*. Region of residence*. 1 = Eastern China, 2 = Central China, 3 = Western China*.*

***County-level SES:*** The county-level SES was indicated by the average years of education in the county, ranging from 6.8 years to 12.19 years. The data was derived from the Sixth National Population Census (2010).

#### Analytical strategies

The hypotheses were tested with structural equation modeling using Mplus 8.0. Data were missing for parental involvement (T1_*missing*_ = 2.55–4.83%, T2_*missing*_ = 2.33–3.24%), academic performance (T1_*missing*_ = 2%, T2_*missing*_ = 1.39%), and other demographic covariates (0.03–15.4%). Before the main analysis, attrition analysis was conducted using logistic regression. No evidence suggested that attrition was due to parental involvement and academic performance differences. Thus, missingness was handled using maximum likelihood estimation (ML) with the assumption of Missing at Random.

First, Confirmatory Factor Analysis was conducted to examine the factor structure of the construct of parental involvement across time, and the model fit for each point was assessed separately. The commonly used Goodness of Fit Index was used to determine the fit of the model, including Chi-Square Goodness, comparative fit index (CFI), Tucker–Lewis index (TLI), Root Mean Square Error of Approximation (RMSEA), and Standardized Root Mean Square Residual (SRMR). Following Hu and Bentler (1999)’s suggestions, the model fit was considered good—CFI > 0.95, TLI > 0.95, RMSEA < 0.06, SRMR < 0.08. Then the longitudinal measurement invariance of parental involvement was examined to ensure the equivalence across time. A set of nested comparison tests were conducted corresponding to configural, metric, scalar, and strict levels of invariance. Measurement invariance was considered strong if the configural, metric, and scalar levels were invariant.

In addition, the data structure is nested in nature, with students nested within schools and schools nested within communities, but all variables of interest were at the individual level. Therefore, instead of constructing multi-level models, a set of covariates at student-, family-, school- and community levels were included, and maximum likelihood estimation with robust standard errors method was used to account for the non-dependency of observations in the analysis. Cross-lagged models were adopted to examine the reciprocal hypotheses between parental involvement and adolescents’ academic performance. Compared to traditional cross-sectional regression approaches, the cross-lagged model can rule out alternative explanations of findings with the control of variables measured earlier and established time order between variables. Each outcome variable (parental involvement and students’ academic performance) was regressed on its auto-regressor and cross-lagged on the other variable in the prior time point. For example, students’ academic performance in 8th grade regressed from the 7th academic performance and parental involvement in 7^th^ grade. Last, multi-group SEM with Wald Chi-Square test was adopted to examine whether the hypothesized reciprocal relationship varies across students’ genders. The study used the benchmarks 0.03 (small effect), 0.07 (medium effect), and 0.12 (large effect) to interpret the size of cross-lagged coefficients (Orth et al., 2022).

## Results

### Descriptive Statistics

Table 1 presents the descriptive information of variables of interest and gender differences. In general, over 90% of participating students were ethnic Han, with half of them living with their parents and residing in urban areas. Parents were significantly more involved in their daughters’ learning through educational trips, education discussion, and behavior control, while more involved in their sons’ learning through initiating school contact and participation. Compared to boys, girls gained significantly higher scores in their midterm standard tests in 7th grade and 8th grade.

**Table 1:** Descriptive statistics of parental involvement, academic performance, and other covariates

		Boys *N* = 4842	Girls *N* = 4436	*t* (9276)
	Range	Mean (SD) / %	Mean (SD)/ %	
Covariates				
Ethnicity (Han, yes) %	0–1	91.94	91.05	
Only child status (yes)%	0–1	47.44	42.87	
Living with parents (yes)%	0–1	76.15	77.61	
Family SES	−1.38–2.33	0.02 (0.78)	0.01 (0.79)	0.56
School type (public, yes) %	0–1	92.19	95.06	
School ranking (the highest tier, yes) %	0–3	22.18	22.57	
Place of residence (urban, yes) %	0–1	47.03	48.62	
Region of residence (East, yes) %	0–3	56.15	55.52	
County-level SES	6.80–12.19	9.54 (1.43)	9.57 (1.44)	0.41
Parental Involvement				
T1-ET	1–6	2.21 (1.29)	2.23 (1.25)	−0.57
T2-ET	1–6	2.07 (1.02)	2.13 (1.02)	−2.69**
T1-ED	1–3	2.31(0.50)	2.35 (0.51)	−3.64***
T2-ED	1–3	2.20 (0.53)	2.24 (0.55)	−4.18***
T1-BC	1–3	2.36 (0.40)	2.40 (0.39)	−4.50***
T2-BC	1–3	2.28 (0.40)	2.33 (0.40)	−5.97***
T1-SCP	0–4	0.79 (0.41)	0.71 (0.42)	8.81***
T2-SCP	0–4	0.79 (0.42)	0.73 (0.42)	6.66***
Academic Performance				
T1-MS	55.49–293.93	204.94 (26.51)	217.77 (21.95)	−25.02***
T2-MS	116.46–257	205.02 (27.72)	217.09 (23.12)	−22.53***

*T1* = Time 1 (7th grade), T2 = Time 2 (8th grade)

*ED* education discussion, *ET* educational trips, *BC* behavioral control, *SCP* school contact and participation, *MS* Midterm test score

*p < 0.05. **p < 0.01. ***p < 0.001

### Testing Longitudinal Invariance of Parental Involvement

Before constructing the final Cross-lagged model, this study first tested the factor structure of latent construct parental involvement across time points to ensure the construct validity. CFA demonstrated a four-factor model of parental involvement. Standardized factor loadings were satisfactory and significant except for one item, “Communicate about physical health” with a factor loading of around 0.3 in two waves. Therefore, the item was removed in the further analysis. As the final CFA model presented in Table 2, standardized factor loadings for 17 items were significantly associated with latent variable parental involvement ranging from 0.47 to 0.88 (*p* < 0.001). According to the rule of thumb, all factor loadings in the CFA model at two-time points were considered as fair (>0.45) to excellent (>0.71). Good model fits were achieved at two-time points (7^th^ grade: χ^2^_100_ = 942.19, *p* < 0.001, CFI = 0.98, TLI = 0.98, RMSEA = 0.03, SRMR = 0.02; 8^th^ grade: χ^2^_100_ = 1189.13 *p* < 0.001, CFI = 0.98, TLI = 0.97, RMSEA = 0.03, SRMR = 0.02).

**Table 2:** Standardized factor loadings for the latent construct of parental involvement

	7th Grade				8th Grade			
Items of constructs	ED-1	ET-1	BC-1	SCP-1	ED-2	ET-2	BC-2	SCP-2
The relationship between he/she and his/her friends	0.73				0.79			
Things happened at school	0.77				0.83			
The relationship between he/she and his/her teachers	0.74				0.80			
His/her feelings	0.66				0.74			
Visiting museums, zoos, science museums, etc		0.88				0.77		
Going out to watch movies, shows, sports games, etc		0.88				0.83		
Time he/she spends on watching TV			0.51				0.52	
Time he/she spends on the Internet			0.66				0.71	
His/her behavior at school			0.55				0.61	
His/her dress style			0.54				0.54	
His/her homework and examination			0.52				0.58	
Whom he/she makes friends with			0.57				0.58	
Teacher-parent communication frequency				0.78				0.74
Communication foci of schoolwork				0.58				0.64
Communication foci of the child’s morality				0.60				0.58
Communication foci of mental health				0.71				0.68
Communication foci of friends of the child				0.47				0.50

All factor loadings were significant at *p* < 0.001 level

*ED* Education Discussion, *ET* Educational Trips, *BC* Behavioral Control, *SCP* School Contact and Participation

Then the longitudinal invariance of parental involvement across time was tested to ensure the measurement equivalence. In the configural model, all parameters were freely estimated and exhibited good model fit (CFI = 0.97, TLI = 0.97, RMSEA = 0.03, and SRMR = 0.02). Then factor loadings were constrained to be equal across time to test for the metric invariance (CFI = 0.97, TLI = 0.97, RMSEA = 0.03, and SRMR = 0.03). Scalar and strict invariance were also tested and presented in Table 3. Although Chi-square test difference is the most commonly used modification index to compare the measurement invariance models, it is highly sensitive to the large sample size. As in this study, a large sample size led to a significant Δχ^2^ value when comparing measurement invariance models. Therefore, this study used alternative fit indices CFI, RMSEA, and SRMR to examine the measurement invariance. A change of ≤0.01 in CFI, a change of ≤0.01 in RMSEA, and a change of ≤0.03 in SRMR favored higher levels of measurement invariance (Chen, 2007). As shown in Table 3, all indices supported the establishment of strict invariance, providing strong evidence of reliability and validity of measurement across time.

**Table 3:** Longitudinal measurement invariance model fit statistics of parental involvement

Model	χ^2^	df	CFI	TLI	RMSEA	SRMR	Model Comparison	∆χ^2^	∆df	*p*
1.7th Grade	942.19	100.00	0.98	0.98	0.03	0.02				
2. 8th Grade	1189.13	100.00	0.98	0.97	0.03	0.02				
3. Configural invariance	3318.75	456.00	0.97	0.97	0.03	0.02				
4. Metric invariance	3402.77	469.00	0.97	0.97	0.03	0.03	4 vs. 3	84.03	13.00	***
5. Scalar invariance	4301.40	486.00	0.97	0.96	0.03	0.03	5 vs. 4	898.63	17.00	***
6. Strict invariance	4937.58	490.00	0.96	0.95	0.03	0.04	6 vs. 5	636.18	4.00	***

*p < 0.05. **p < 0.01. ***p < 0.001

### Testing Association between Parental Involvement and Adolescents’ Academic Performance

To examine the bidirectional relationship between parental involvement and adolescents’ academic performance, cross-lagged models were constructed through SEM approach. Specifically, this study followed the following steps to compare the SEM models: (1) M1: a model with only auto-regressive paths from 7th grade to 8th grade, indicating test-retest stability; (2) M2: a model with both auto-regressive paths and cross-lagged paths from parental involvement at 7th grade to academic performance at 8th grade, supporting an parents-driven hypothesis; (3) M3: a model with both auto-regressive paths and cross-lagged paths from academic performance at 7th grade to parental involvement at 8th grade, supporting a child-driven hypothesis; (3) M4: a model with auto-regressive paths and all cross-lagged paths, representing a reciprocal relationship. Goodness-of-fit indices of the nested four models were used to determine which model best fit. Overall, the four models all had good model fits (CFI = 0.97, RMSEA = 0.03, SRMR = 0.03–0.04). As the Chi-square differences among M4 and other models were all significant (*p* < 0.01), indicating M4 was the best-fitting model, it supported the hypothesized reciprocal relationship.

**Fig. 2:**
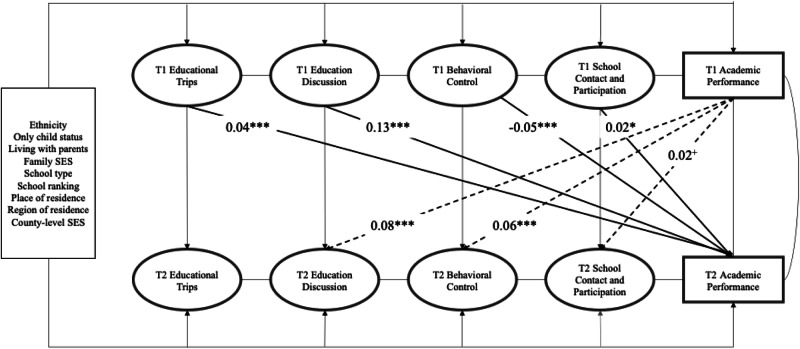
Standardized Estimates for Cross-Lagged Model for the Associations between Different Forms of Parental Involvement and Academic Performance. *Note*. T1 = Time 1 (Grade 7), T2 = Time 2 (Grade 8). Bold paths indicate the cross-lagged influence of parental involvement in 7th grade on academic performance in 8th grade. Dashed paths indicate the cross-lagged influence of adolescents’ academic performance in 7th on parental involvement in 8th grade. Only significant paths were presented. For simplicity, correlation coefficients at the same time points were not presented. +*p* < 0.1, *p < 0.05. **p < 0.01. ***p < 0.001

Figure 2 presents the final model representing the reciprocal association between parental involvement and young adolescents’ academic performance. After controlling for county-, school-, family-, and individual-level covariates, from a parent-driven perspective, parental involvement in 7th grade predicted adolescents’ midterm test scores in 8th grade. Specifically, a higher frequency of educational trips (*β* = 0.04, *p* < 0.001), education discussion (*β* = 0.13, *p* < 0.001), and school contact and participation (*β* = 0.02, *p* < 0.05) in 7th grade predicted better academic performance in 8^th^ grade. More behavioral control was negatively associated with later academic performance (*β* = −0.05, *p* < 0.001). From a child-driven perspective, adolescents’ academic performance in 7^th^ grade predicted two aspects of parental involvement in 8^th^ grade, namely, education discussion (*β* = 0.08, *p* < 0.001) and behavioral control (*β* = 0.06, *p* < 0.001). Sensitivity analyses were conducted to examine the robustness of the findings by substituting the midterm grades with CAT. The findings generally aligned with the original models using midterm grades.

### Moderation Effect of Gender

**Fig. 3:**
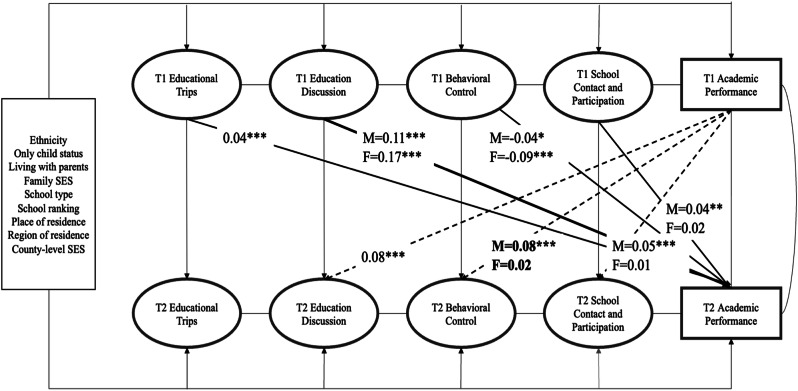
Gender Multigroup Comparison in the Reciprocal Association Between Parental Involvement and Adolescents’ Academic Performance (Standardized Solution). *Note*. T1 = Time 1 (Grade 7), T2 = Time 2 (Grade 8). Path coefficients indicate the standard coefficients from multigroup SEM analysis (M = male adolescents, F = female adolescents). Wald Chi-square tests suggested only the coefficients from T1 academic performance to T2 Behavioral Control differed significantly for boys and girls. Bold paths indicate the cross-lagged influence of parental involvement in 7th grade on academic performance in 8th grade. Dashed paths indicate the cross-lagged influence of adolescents’ academic performance in 7th on parental involvement in 8th grade. Only significant paths were presented. For simplicity, correlation coefficients at the same time points were not presented. *p < 0.05. **p < 0.01. ***p < 0.001

Multi-group SEM approach was applied to examine the extent to which observed effects varied by gender. Wald Chi-square test was conducted to examine whether differences in the coefficients between models of boys and girls were significant. As shown in Fig. 3, different forms of parental involvement in 7th grade were significantly associated with boys’ and girls’ academic performance in 8th grade with varying magnitudes. Educational trips (*β*_*boy/girl*_ = 0.04, *p* < 0.001) and education discussion (*β*_*boy*_ = 0.11, *p* < 0.001; *β*_*girl*_ = 0.17, *p* < 0.001) positively predicted academic performance both for boys and girls, while school contact and participation was significantly associated with academic performance only for boys (*β*_*boy*_ = 0.04, *p* < 0.01) but not girls (*β*_*girl*_ = 0.02, *p* > 0.05). Behavior control negatively predicted later academic performance both for boys and girls (*β*_*boy*_ = −0.04, *p* < 0.05; *β*_*girl*_ = −0.09, *p* < 0.001). Academic performance in 7th grade significantly predicted education discussion for both boys and girls (*β*_*boy/girl*_ = 0.08, *p* < 0.001). In contrast, academic performance only significantly predicted behavior control for boys (*β*_*boy*_ = 0.08, *p* < 0.001), but not for girls (*β*_*girl*_ = 0.02, *p* > 0.05). Similarly, academic performance significantly predicted parents’ school contact and participation for boys (*β*_*boy*_ = 0.05, *p* < 0.001), but not for girls (*β*_*girl*_ = 0.01, *p* > 0.05). Despite the magnitude of coefficients in multi-group models being different between gender groups, the Wald Chi-square test indicated only coefficients in the path from academic performance to behavior control differed significantly for boys and girls, suggesting parental involvement generally exhibiting equivalent impacts for boys and girls.

## Discussion

A growing body of research has underscored the indispensable role of parental involvement in adolescents’ academic performance. As entering puberty, young adolescents are confronted with greater academic demands and pressure. Such change impels parents to adjust their ways of responding. However, extant studies have accorded less attention to understanding the child-driven influences on parental involvement. From the perspective of transactional theory, this study has examined the reciprocal relationships between parent-reported involvement and adolescents’ academic performance based on two-year nationally representative longitudinal data in China. It was found that the degree to which and how parents were involved in learning was reciprocally associated with young adolescents’ academic performance after controlling for individual-, family-, school-, and community-level covariates. The varied magnitudes of the associations between different forms of parental involvement and parental involvement indicate the multidimensionality of parental involvement and their unique contributions to adolescents’ academic performance. Meanwhile, gender variation was found in the forms of parental involvement and their unique associations with adolescent’s academic performance.

### Autonomy-Supportive Parental Involvement Predict Higher Academic Performance

Consistent with findings from the most recent meta-analysis review (Barger et al., 2019), parental involvement was generally associated with young adolescents’ academic performance from 7th to 8th grade with small to large effect sizes. The generally positive effect of parental involvement echoes Coleman’s report (1966) that parental involvement explains a large proportion of variance in children’s academic performance. Meanwhile, this study found that different forms of parental involvement uniquely contributed to adolescents’ academic performance after controlling for individual-, family-, school-, and community-level covariates. Among the four forms of parental involvement measured, autonomy-supportive parental involvement was found to be positively associated with young adolescents’ academic performance.

Specifically, education discussion was identified as the strongest predictor of later academic performance. This finding is consistent with previous studies that discussing school life and learning consistently benefits adolescents’ academic life, especially for young adolescents at middle school age (Hill & Tyson, 2009). Further, previous studies generally consider the positive role of educational trips for early childhood children (Boonk et al., 2018). This study expands the discussion that educational trips can similarly benefit young adolescents’ later academic performance. In contrast to previous studies that attribute the null effect of parent-teacher communication to the parent-teacher loosely connected relationship in the Chinese context (Huntsinger & Jose, 2009), this study found that the frequency of parent-initiated school contact and participation positively predicted young adolescents’ academic performance. One possible explanation would be the changing landscape of parent-teacher communication with the rapidly increasing use of communication apps, such as WeChat and DingTalk, enabling Chinese parents to connect unprecedentedly with teachers and school personnel (Gong et al., 2021).

Not all forms of parental involvement were found to have positive influences on young adolescents’ academic performance. In particular, when parental involvement was narrowly defined as behavioral control of young adolescents’ lives and learning through restrictions and supervision, it constituted small but significantly negative impacts. Although previous studies suggested that behavioral control is less concerning than psychological control, similar negative impacts of behavioral control have been observed mostly in Western countries, especially in the reading and mathematics homework context (Dumont et al., 2014; Silinskas & Kikas, 2019). Compared to Western countries, Asia countries like China are long perceived to have inherited a culture emphasizing relational interrelatedness and parental authority. Thus, parents’ control behaviors will bring less, if not zero, negative influences on adolescents (Wang et al., 2007). Findings from this study, keeping up with the most recent research (Peng et al., 2023), found that parents’ behavioral control negatively influenced Chinese adolescents.

The latest finding suggests a different path from an earlier work that parents’ behavioral control enhanced Chinese adolescents’ academic performance (Wang et al., 2007). The discrepancy may be due to the changing societal norms compared to millennium China. Although behavioral control is culturally and inherently endorsed or conceded in Chinese culture, young adolescents nowadays also attach great importance to their personal and behavioral autonomy. The resentment brought by parents’ behavioral control will lead to less desired academic performance. An alternative explanation would relate to how parents’ behavioral control was operationalized and measured. This study focused on parental restrictions on their children’s behaviors and whereabouts, while Wang et al. (2007) also took parental solicitation, an active effort to obtain children’s information, into account. Despite the discrepancies, the finding pertinent to the negative influence of behavioral control suggests that the need for autonomy is universal. Parental involvement is not always effective if it puts too much pressure on children through behavioral control or undermines children’s autonomy.

Given the varied and specific impacts of different forms of parental involvement, Chinese parents are advised to adopt more child-centered and autonomy-supportive forms of parental involvement in their children’s learning, e.g., initiating daily communication about their children’s school life, taking children for educational trips to museum, movies, and sports center, and closely connected with teachers and school personnel. Meanwhile, one shall not attribute the sole responsibilities to parents and be aware of the roles of teachers, schools, and communities. This is certainly true in the case of educational trips. If the space, infrastructure, and facilities in the neighborhood were not accessible and affordable, parents would not have the opportunity to take their children to museums or theaters. In such a case, local governments and communities play indispensable roles in creating an enabling environment for families, especially those underprivileged families. Schools and teachers should also develop more participatory opportunities to keep the communication channels open and available for all.

### The Better Academic Performance, The More Parental Involvement

This study further discusses how adolescents’ academic performance has shaped the way parents are involved in their children’s learning. It was found that 7th-grade academic performance significantly predicted the extent of education discussion and behavioral control in the 8th grade, suggesting that parents adjust their involvement in response to their children’s academic performance. One unexpected pattern from the finding is worth noting: parents increased their levels of education discussion and behavioral control for children who had better academic performance in 7^th^ grade. This is contradicted by the reactive hypothesis that parents tend to increase their levels of involvement when children show less desired performance at school, as parental involvement can be of any complementary practices (Dumont et al., 2014).

One of the possible explanations is related to parents’ self-efficacy toward parental involvement. When children are doing well at school, parents may feel confident about their parenting strategies, and thus, they are more motivated to increase subsequent engagement (McNeal, 2012). In return, academic success inspires parents to mobilize higher levels of actual resources, commitment, and involvement. Another possible explanation for this might be concerned with the declining intergenerational mobility in China. With the fierce competition for high-quality education escalating, education, as the ladder of opportunity, has become much more challenging to climb on — because of intensive competition for seats in high-performing schools. Facing the pressure of the “rat race,” Chinese parents feel compelled to sustain their children’s academic edge through increased involvement, even when their children are already outperforming their peers. This trend has also been corroborated by one recent study in urban China (Xiong et al., 2021). Given the essential role of parental involvement in adolescents’ academic performance, it is vital to motivate parents of low-performing students to actively engage in their children’s learning through autonomy-supportive involvement. Teachers and schools are the crucial conduits for imparting effective parental involvement strategies, thus enhancing parents’ confidence to engage in their children’s learning and school life actively.

### Gendered Parental Involvement

This study discovered distinct parental involvement patterns with early adolescents based on gender. Parents tended to be more directly involved in their daughters’ learning by initiating educational trips and discussions. In contrast, they were more inclined to seek frequent communication with teachers and engage in school activities regularly to support their sons. This is because girls are more concerned about their relationships with others, which makes them more likely to seek social support than boys, especially when they are exposed to academic stress and demands, while boys, who generally underperform compared to girls, their parents may want to seek out more for teachers’ support (Borgonovi & Montt, 2012).

Inconsistent with previous studies in high-income contexts (Dumont et al., 2014; Hong et al., 2010), Chinese parents tended to impose higher levels of behavioral control for girls than boys during the onset of puberty. Usually, it is assumed that boys’ more externalizing behaviors may elicit parents’ higher levels of control (Wang et al., 2011). Yet, the findings from the study indicated that Chinese parents still follow gender-based scripts either consciously or unconsciously, exhibiting more restrictions on their daughters. According to gender schema theory, gendered behavioral control reflects the persistence of traditional gender expectations in contemporary China: girls should adhere to traditional standards of obedience and propriety (Zhang & Ng, 2022).

Despite gender-based differences in parental involvement, this study found that the impacts of different forms of parental involvement on academic performance were comparable for boys and girls. This contributes to mixed research findings and underscores that young adolescents, irrespective of gender, benefit from autonomy-supporting parental involvement (Dumont et al., 2014). They are equally negatively affected by excessive behavioral control during puberty’s transitional phase. This again signifies the need for autonomy is universal for young adolescents.

Moreover, parents adjusted their involvement strategies slightly differently in response to their children’s academic performance in 7th grade. Similarly, parents would initiate more conversations about school life in 8th grade with their children who had already shown better academic performance in 7th grade. Dissimilarly, boys’ better performance in 7th grade would lead to parents’ higher levels of behavioral control but not for girls. The finding does not imply that parents exhibited more behavioral control for boys. It is worth noting that the increase in control for high-performing boys does not imply that it surpassed the already higher levels imposed on girls. On the contrary, such differences can be explained by the persistent and stereotypical gender expectations in China that girls are ‘passive’ and boys are ‘active’ (Evans, 1997). Unlike boys, girls, regardless of their academic performance, should be mature, submissive to their parents’ authority, and confined by social rules and norms. For boys, disobeying and breaking the rules is far more acceptable and even taken as a merit for being proactive, adventurous and courageous (Mesman & Groeneveld, 2018).

As young Chinese adolescents have experienced gendered parental involvement, they may internalize the implicit socially desirable gender-appropriate behaviors (e.g., girls should be obedient, subordinate, and submissive; boys should not reveal their feelings to others), which is ultimately detrimental to gender equality and their development. As important agents of socialization, parents should increase awareness about gender equality. This could be done by leveraging the power of social media to challenge the stereotypical gender norms. Parent education programs should also incorporate gender-responsive parenting strategies and resources to address parents’ practical concerns.

### Limitations and Future Research Directions

There has been extensive discussion about the influence of parental involvement on academic performance using cross-sectional data. Most studies have focused on certain aspects of parental involvement and used composite scores to indicate parental involvement, while in nature, parental involvement is a multidimensional construct. Thus, it is not clear which aspects of parental involvement are more predictive and how children’s characteristics shape parental involvement in this interactive process. This study addresses the research gap by examining the reciprocal relationship between different forms of parental involvement and adolescents’ academic performance using national representative and longitudinal data in a Low- and Middle-Income context.

Some caveats of this study should be noted. First, parental involvement is a multidimensional construct with abundant extensions. Despite this study conceptualizing parental involvement broadly and using multiple indicators, it is still not sufficient to capture the full picture and uniqueness of Chinese parental involvement, given the limited number of items in a nationwide survey. Future research should address this limitation by developing a comprehensive and context-specific tool to measure parental involvement. Second, although the two-wave longitudinal design is better than the cross-sectional design for building up clear temporal orders among variables and sufficient for the test of the reciprocal hypothesis, the two-wave longitudinal design cannot sufficiently capture the pattern of change between parental involvement and academic performance in the transitional period of puberty. Future studies can adopt three or more wave designs to delineate a comprehensive developmental trajectory. Third, in-depth case studies are needed to understand the mechanism of parent involvement. Given the limitation of quantitative studies, this study can only provide plausible interpretations of our findings. For example, why do Chinese parents intend to increase their involvement in children’s learning when their children outperform their peers? Does parent’s gender influence their ways of gender socialization regarding academic learning? Future studies may further delve into these unique findings.

## Conclusion

Parental involvement plays a paramount role in adolescents’ academic learning. Yet, previous studies predominately have focused on the influences of parental involvement and treated it as a broad construct. The present study utilized nationally representative longitudinal data to address the reciprocal relationships between different forms of parental involvement and adolescents’ academic performance. It highlights that autonomy-supportive involvement strategies benefit Chinese adolescents’ academic performance during early adolescence, where children’s autonomy is not prioritized when it comes to academic learning. Further, the findings reveal that parents respond differently to their children’s performance at school with significant gender differences. It argues that Chinese adolescents are in great need of autonomy-supportive, responsive, and gender-sensitive support from their parents and caregivers equally as their counterparts in other socio-cultural contexts.
